# Age related differences in phylogenetic diversity, prevalence of Shiga toxins, Intimin, Hemolysin genes and select serogroups of *Escherichia. coli* from pastured meat goats detected in a longitudinal cohort study

**DOI:** 10.1186/s12917-020-02479-0

**Published:** 2020-07-30

**Authors:** Eunice Ndegwa, Aber Alahmde, Chyer Kim, Paul Kaseloo, Dahlia O’Brien

**Affiliations:** 1 Agricultural Research Station, Virginia State University, Petersburg, VA 23806 USA; 2 Department of Biology, Virginia State University, Petersburg, VA 23806 USA; 3 College of Agriculture, Virginia Cooperative Extension, Virginia State University, Petersburg, VA 23806 USA

**Keywords:** Goats, Age, Cohort, Virulence genes, Phylogenetic grouping, *E. coli*, Serotype

## Abstract

**Background:**

Little is known on significance, diversity and characteristics of gut *E. coli* in goats despite their importance as food animals globally. We characterized the temporal dynamics in diversity of *E. coli* in fecal samples from a cohort of goat kids and adult meat goats on pasture over a one-year period. Isolates were characterized based on phylogenetic grouping, virulence genes; shiga toxins 1 and 2 (*Stx1&Stx2*) (STEC), intimin *(eaeA),* hemolysin (*hly)* and select important sero-groups (026, 045, 0103, 0126 and 0146) using molecular methods.

**Results:**

A total of 516 *E. coli* isolates were screened. Prevalence of virulence genes and STEC was 65 and 56% respectively. Prevalence of virulence genes and STEC was significantly higher in goat kids less than six months (76% /66%) than adults (48% /28%). Isolates with virulence profiles of two or more genes were also higher in young goat kids (50%) than adults (20%). Entero-pathogenic *E. coli* (EPEC-*eaeA* gene only) were mostly from pre-weaned goat kids while *hly* gene only isolates were significantly higher in adults. The *stx1*, *stx2* and *hly* genes peaked around weaning (60, 63 and 52%) respectively. Goats kids were mostly hosts to group D (59%) while adults older than one year had B1 (75%) isolates. Group D isolates were most abundant at weaning (64%) and diarrhea samples (74%). Group B2 isolates overall (6%) were mostly detected around weaning (63%) while A isolates were 4% overall. Twenty-four isolates belonged to sero-groups 026, 0103 and 0146 with 70% of the isolates detected around weaning. Nineteen of these isolates were STEC with most harboring the *stx1/stx2/hly/eae* (25%) profile. Most belonged to O26 sero-group (75%) and phylogroup D (75%).

**Conclusion:**

To our knowledge this is the first study to highlight longitudinal age related differences in *E. coli* phylogenetic diversity, abundance of virulence genes and select important sero-groups in goats. Differences detected suggest a possible role of age and weaning stress in influencing *E. coli* diversity in the gut of goats. The findings are relevant to both animal and public health to advise on further studies on caprine *E. coli* isolates as animal and human pathogens.

## Background

Goats are an important source of meat and milk globally and are also gaining popularity in North America as a meat source due to an increased migrant demand [1]. However little is known about the diversity of *E. coli* in the gastrointestinal tract of goats including their potential as pathogens. *E. coli* is associated with a number of disease conditions in animals and humans. In ruminants, bacterial scours during pre-weaning and peri-weaning period are often caused by *E.coli* as primary pathogen or in complex interactions with parasites and viruses [2]. A key role of *E. coli* in neonatal diarrhea in goat kids was demonstrated by the reduced incidence of diarrhea in offspring when nursing does were vaccinated against *E. coli* during pregnancy [3]. *E. coli* pathotypes include enteric strains: enteropathogenic (EPEC), enterohemorrhagic (EHEC), enterotoxigenic (ETEC), enteroaggregative (EAEC), enteroinvasive (EIEC) and diffusely adherent (DAEC). Strains that cause extra-intestinal infections include those that cause urinary tract disease and meningitis and are generally referred to as ExPEC [4]. The ETEC strains are associated with diarrhea in young calves [5] while the EPEC strains whose characteristic hallmark is the attaching and effacing (A/E) lesion on the intestinal brush border are commonly detected in both healthy and diarrheic animal fecal samples [6]. They are also involved in causing diarrhea in young children. The ability to form the A/E lesions is accorded by the intimin protein coded by the *eaeA* gene [7, 8]. Pathogenic *E. coli* utilizes a number of virulence factors to colonize and cause disease in animals and humans among which, shiga toxins 1 and 2 (Stx1, Stx2), intimin (enterocyte attaching and effacing protein (*eaeA*) and hemolysin are well known and widely studied [9]. The shiga toxin producing *E. coli* (STEC) produce either shiga toxin 1, shiga toxin 2 or both and the genes are carried by bacteriophages [10]. Additionally they produce other virulence molecules that determine their pathogenicity in different host tissues [9]. The role of STEC in causing diseases in ruminants, the predominant host reservoirs [8] and also other farm animals is not clearly understood. They have been isolated in feces from both healthy and diarrheic animals [8, 11]. On the other hand, STEC strains are important human pathogens and includes strains that cause bloody diarrhea, non- bloody diarrhea and urinary tract infections in humans (EHEC) [8]. The most studied of this pathotype is the O157:H7 serotype although the importance of other non-157 STEC in human disease is now widely acknowledged [12–14]. Both shiga toxin 1 and 2 inhibit cell protein synthesis by cleaving the ribosomal RNA resulting in cell death [4, 10]. While the shiga toxin 1 is highly conserved in shiga toxin –producing *E.coli* (STEC), the shiga toxin 2 is highly variable with some variants being more associated with higher virulence in humans [15]. The importance role of non-O157 *E.coli* STEC strains in public health has recently been reviewed [16–18]. Ruminants including cattle, sheep and goats are known to be reservoirs to *E. coli* isolates of public health importance including STEC O157 and non-O157 strains [7]. Some non-O157 STEC serotypes (026, 045) from goats have also been associated with diarrhea in people [17, 19]. Acknowledging the importance of STEC in public health, Europe now requires and emphasizes meat inspection efforts to ensure absence of STEC from slaughtered small ruminants [20]. Only a few studies have characterized prevalence of virulence genes in *E.coli* isolates from goats and sheep [21–26]. Moreover, prevalence of these important *E. coli* strains (STEC, EPEC) and their virulence gene profiles have not been investigated in US sheep and goat flocks despite the increasing number of meat goats in the US for the last decade and existence of several dairy goat flocks and also sheep flocks. Additionally, sheep and goats are almost always part of livestock found in petting zoos, state fairs and also companion animals in some US homes. Thus these animals interact with people closely and also with the environment as most are reared in pastures. Goats have been incriminated in human non O157 *E.coli* outbreaks (O45) through contact [17] and recent study in meat goats in a US slaughter house detected a number *E.coli* serotypes in fecal samples that are of significance to public health underscoring the need for more studies targeting meat goats [27].

In studies from other countries in Europe the findings on the prevalence of important *E.coli* virulence genes differ from study to study [21, 24], different geographical locations, different ages, different seasons [28] and species of animals. In cattle and dairy goats, the pattern of expression of the different virulence genes by *E. coli* has been shown to differ based on age of the host. Stress has also been shown to cause increased prevalence of certain virulent strains including O157 in cattle [29, 30]. Thus it seems that colonization and shedding of important *E.coli* strains in a host may be dependent on many factors that have yet to be fully described including age, geographic location, stress, management factors, and production system among others [31].

*E.coli* are generally characterized into four phylogenetic groups that include A, B1, B2 and D based on the presence of the *chuA* and *yjaA*, and the anonymous DNA fragment *Tspe4.C2* [32, 33]. Studies have shown that the phylogenetic groups differ in the ecological niches they predominantly inhabit, size of genomes, presence of virulence factors and in some cases the presence of antibiotic genes. In particular, the virulent extra-intestinal serotypes are mostly found in group B2 and often in group D whereas, majority of the commensal serotypes fall in group A and B1. Intestinal pathogenic strains are mainly the phylogenetic groups A, B1 and D (reviewed in [34]).

With limited information on the diversity of gut commensal *E. coli* in meat goats and their significance as pathogens, this study was designed with the objective of characterizing the temporal dynamics in *E. coli* phylogenetic distribution, prevalence of select virulence genes and select important serotypes in pastured meat goats of different age groups.

## Results

### Prevalence of virulence genes in pre-weaned goat kids and nursing does

**Fig. 1:**
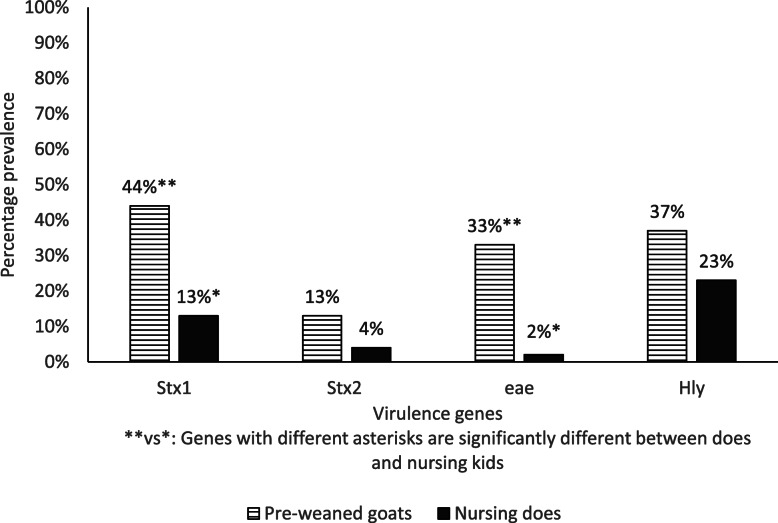
Comparison of virulence genes in *E. coli* from healthy pre-weaned goats and nursing does

All goat kids and nursing does were apparently healthy at the time of sampling and grazing together on pasture. In total, 128 *E. coli* isolates that included 75 and 53 isolates from goat kids and does respectively were screened for virulence genes over the three sampling points before weaning. The virulence genes *stx1, hly*, and *eaeA* were detected in goat kids as young as 3 weeks old and were detected at each of the three sampling points. In nursing does, only the virulence genes *stx1* and/or *hly* were detected at all the three sampling points*.* The *stx2* gene was detected in very few isolates from goat kids during the same period but none of the isolates harbored the enterohemorrhagic *Escherichia coli* (EHEC) type *Stx2* gene. In young goat kids the prevalence of *stx1* ranged from 30 to 58%, *stx* 2; 4–29% *eaeA*; 7–71% and *hly* 22–63%. In nursing does the prevalence of the genes *stx1* ranged from 10 to 21%, *stx 2* variant 0–20%, *eaeA* 0–5% and *hly* 14–29%. For both *stx1* and *eaeA* genes, the prevalence in *E. coli* isolates from pre-weaned goat kids was significantly higher (*P* = 0.0002 and *P* < 0.0001) respectively than those from the nursing does (Fig. 1). It was interesting to note that a high percentage of *E. coli* isolates from apparently healthy 3-week goat kids harbored the *eaeA* gene (71%). The mean prevalence of the virulence genes in the two groups during the three sampling points is shown below (Table 1).

**Table 1:** *E. coli* virulence genes screened and number of positives isolates in different pastured goat age groups

	Pre-weaned kids	Nursing does	Weaning period (kids)	Six months	Yearling	All adults	Diarrhea
Virulence genes	*n* = 75	*n* = 53	*n* = 163^b^	*n* = 66	*n* = 41	*n* = 102^a^	*n* = 47
*Stx1*	30 (40)	7 (13)	99 (61)	30 (45)	13 (31)	27 (26)	23 (49)
*Stx2 (*EHEC type*)*	0	0	24 (15)	0	0	0	8 (17)
*Stx2 (other)*	10 (13)	2 (4)	101 (62)	12 (18)	13 (31)	7 (7)	26 (55)
*EaeA*	25 (33)	0	29 (22)	0	0	2 (2)	3 (6)
*Hly*	28 (37)	12 (23)	74 (45)	29 (44)	13 (31)	33 (32)	17 (36)

^a^includes the nursing does isolates; () values in brackets represent percentages. ^b^ value in () in this column represent the mean prevalence of the four sampling days

### Prevalence of virulence genes during weaning period

**Fig. 2:**
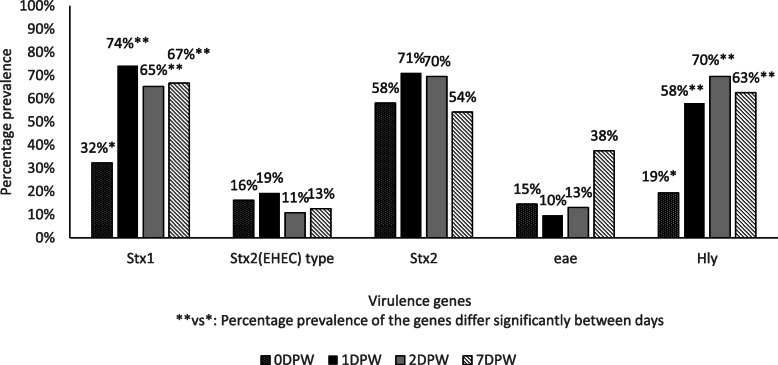
Prevalence of virulence genes in *E. coli* isolates around weaning period

The weaning period is a stressful period in young animals and is often associated with diarrhea in most farm animals. It is unclear if any of the *E. coli* virulence genes evaluated in the study plays a role in colonization of *E. coli* in the gut of ruminants including goats. To evaluate if weaning stress affects the prevalence virulence genes in *E. coli* isolates from young goat kids around the weaning period, we screened 163 *E. coli* isolated from the goat kids during the weaning period (day of weaning (0DPW), one day after weaning(1DPW), two days after weaning (2DPW) and seven days after weaning (7DPW). The four virulence genes were detected in all days sampled during the peri-weaning period (Fig. 2 and Table 1). The three genes *stx1*, *stx2* variant, and *hly* were detected in a high number of *E. coli* isolates at 1DPW, 2DPW and 7DPW. The *Stx1* gene prevalence in these isolates ranged from 65 to 74% while *Hly* ranged from 58 to 70% during the three days post weaning. A unique pattern in which the prevalence of both *stx1* and *hly* genes increased significantly from 0DPW to the other sampling days (1DPW *P* = 0.001), (2DPW *P* = 0.0007) and (7DPW *P* = 0.009) for *Stx1* and hly (1DPW *P* = 0.0001), (2DPW *P* < 0.0001), (7DPW P < 0.0001) was also noted. The highest prevalence of *Stx1* and *hly* genes were detected in *E. coli* isolate at 1DPW (74%) and 7DPW (67%) of isolates respectively. Even at one-week post weaning the prevalence of these two genes was still higher than at the day of weaning (0DPW). Another key finding was the detection of the *E. coli* isolates harboring the *stx2* (EHEC) type gene during this period for the first time in the study. Although detected in a few number of isolates (11–20%), the gene was detected each day during the four sampling points around weaning. The other s*tx2* variant gene was also detected in a high number of isolates during the same weaning period ranging from 54 to 71% of the isolates. The highest prevalence was detected on the first day after weaning (71%) but the gene was still detected in over 50 % of the isolates at 2DPW and one week after weaning.

### Prevalence of virulence genes in post weaned goats and adults

In total, 66 *E. coli* isolates were screened for the virulence genes in the six-month old goats and 42 isolates at one year of age. The *stx1* and *hly* genes were detected in 45% vs 31 and 44% vs 31% of the isolates from six and one year of age respectively. The *eaeA* gene was detected in 2% of the isolates from six months old goat kids while none was detected in isolates from one-year-old goat kids. The *st*x2 variant gene was detected in 18 and 31% of the isolates from six months and one of year age respectively. In adults grazing alongside the cohort of the young growing goats, *stx1* and *hly* were detected in 26 and 32% of the 102 *E. coli* isolates. *Stx2* gene was detected in 7% while *eaeA* were detected in less than 5 % of the adult isolates (Table 1).

### Virulence gene profiles of *E. coli* isolates from different age groups

Of the isolates positive for the virulence genes, the genes were either detected singly or in fourteen different combinations. In general, the prevalence of virulence genes and STEC in kids less than six months was significantly higher than adult goats older than one-year *P* < 0.0001. The highest diversity of isolates based on the virulence gene combinations detected was also in goat kids during peri-weaning [15] and pre-weaning [14] weaning period (Additional file 1) with isolates harboring 2 or more virulence genes being significantly higher (P < 0.0001) than in adult goats. The highest proportion of isolates in the cohort harbored *stx1/stx2/hly* (20%), followed by *stx1/hly (19%)*, *stx2* (15%), *stx1*(10%), *hly* (9%), *stx1/stx2* (7%), *eaeA* (4%), *stx1/stx2/hly/eaeA* (4%)*, stx2/hly* (3%) and *stx1/eaeA/hly (3%)*. The prevalent gene combinations in isolates differed among age groups. In nursing goat kids, most positive isolates harbored a combination of *stx1/hly* (27%) followed by *eaeA* alone (24%) while other virulence gene combinations were detected in less than 10% of the positive isolates. A significantly higher proportion of isolates from adult nursing does and other adults on the farm harbored only the *hly* genes (39%) (*P* < 0.0001) compared to goat kids less than six months while 24% harbored *stx1/hly* genes. *Stx1* and *stx1/stx2* isolates were detected equally in the nursing does group (Additional file 1). Most isolates at around weaning harbored the *stx1/stx2/hly (*29%*)* virulence gene combinations followed by *stx2* (22%) alone and *stx1/stx2* and *stx1/stx2/hly/eae*A both of which were detected in 8% of isolates each. Isolates from 6 months and yearling goats were mostly of the *stx1/hly* profile (39%) and (42%) respectively but a significant numbers of isolates from yearling goats also harbored *stx2* (42%) (Additional file 1).

### Pattern of virulence genes among age groups

**Fig. 3:**
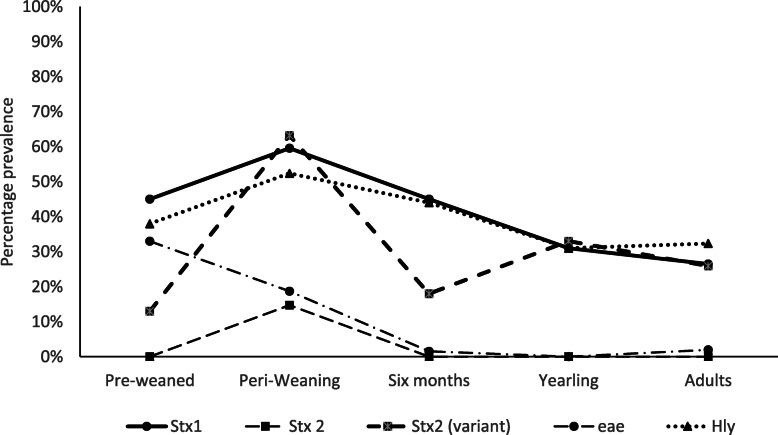
Pattern of prevalence of the *E. coli* virulence genes among age groups

All the isolates from the different age groups of goats were grouped together and the prevalence of the virulence genes in *E. coli* isolates calculated; isolates from pre-weaned goat kids, peri-weaning, six months, yearling and adult animals (pre-weaning and six months later) (Table 1 and Fig. 3. Comparing the different age groups, the highest prevalence and the most diverse virulence genes were detected during the weaning period and the lowest prevalence and diversity was detected in the isolates from goats older than one year. While *hly* and *stx1* were detected in all age groups all the five different genes (*stx1*, *stx2* (EHEC), *Stx2* variants, *eaeA* and *hly)* evaluated were only detected during the weaning period (Fig. 3).

### Phylogenetic diversity of *E. coli* isolates from the meat goats

**Fig. 4:**
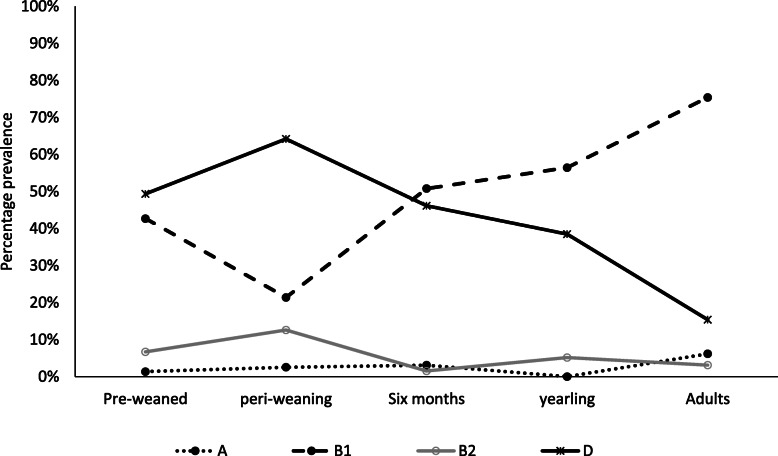
Abundance of the different *E. coli* phylogenetic groups in different age groups

Five hundred and sixteen (516) *E. coli* isolates from the different age groups were genetically characterized using the *chuA*, *yjaA* and DNA fragment *TspE4.C2* as described in materials and methods. The four known phylogenetic groups were detected in all age groups. The predominant *E. coli* phylogenetic groups detected in the cohort were D 239 (46%) and B1 230 (45%) while group B2 [32] and group A [18] were each 6 and 4% respectively (Table 2). In particular, comparing the cohort of pre-weaned nursing goat kids and their respective does who were grazing alongside each other, we found that the predominant phylogenetic groups differed. *E. coli* isolates from goat kids belonged to mostly group D phylogenetic group (49%) and B1 (43%) while those from the nursing does belonged to predominantly group B1 (62%). Twenty-eight (28%) of isolates from the nursing does belonged to group D. Likewise, as a group, *E. coli* isolates from goat kids around weaning belonged mostly to group D phylogenetic group (64%) followed by group B1(21%). There was a significant increase (*P* = 0.02) in the proportion of group D isolates during the weaning period compared to the pre-weaning period. In the six-month cohort of goats, isolates belonged to group B1 (51%) and group D (46%) while isolates from one-year-old goats belonged to group B1 (56%) and group D (38%) (Fig. 4). On the other hand, *E. coli* isolates from adults older than one year belonged to B1 (75%) and group D (15%). An interesting pattern was noted in the prevalence of group D and group B1 phylogenetic groups among the different age groups evaluated (Fig. 4) where group D isolates decreased as the animals grew older while on the other hand group B1 isolates increased with age. Of note was the significantly higher proportions of isolates belonging to group D in pre-weaned goat kids compared to adult animals (*P* < 0.0001) while on the other hand the proportion of group B1 isolates was significantly higher in adult animals (P < 0.0001) than pre-weaned goat kids. Thirty-two [32] isolates belonged to the B2 phylogenetic group. Of these, [25] were isolated from goat kids less than six months while (1, 2 and 4 isolates) were isolated from six months, yearling and adult goats respectively. Group A isolates were less than 10% in all age groups. The highest prevalence of isolates belonging to groups D and B2 in apparently healthy goats were detected during the peri-weaning period (64 and 13%) respectively (Fig. 4 and Table 2). Of the isolates that were also screened for virulence genes, the association between the detected gene combinations and the phylogenetic groups is shown in Additional file 1: Table 1. Overall, most isolates positive for at least one virulence genes belonged to group D (55%), followed by group B1(34%), while group A and B2 were 6 and 5% respectively. Except isolates harboring single genes (*stx1*, *stx2* or *hly*) which belonged to group D and B1 in equal numbers, all other isolates containing two or more virulence genes belonged to group D. Of exception was those isolates with *eaeA* only which predominantly belonged to group D. Isolates belonging to group B2 were distributed in equal numbers in all gene combinations while group A isolates were mostly those that did not harbor the *eaeA* gene (Additional file 1).

**Table 2:** Phylogenetic groups of *E. coli* isolates from different age groups of meat goats and detection of select important serotypes

Age group	Pre-weaned kids	Peri-wean	6 months	Yearling	Does (pre-wean)	Other adults	Diarrhea	Total
Phylo-group	n = 75	*n* = 159	n = 66	*n* = 40	n = 53	*n* = 72	n = 47	*n* = 516
A	1	4	2	2	4	4	1	18 (4)
B1	32	34	33	22	36	62	11	230 (45)
B2	5	20	1	2	1	2	_	31 (6)
D	37	102	30	15	12	4	35	239 (46)
Serotype								
O26	2	14	_	_	_	_	2	18
O45	_	_	_	_	_	_	_	_
O103	1	3	_	_	_	_	_	4
O126	_	_	_	_	_	_	_	_
O146	_	2	_	__	_	_	_	2

() values represent percentages

### Detection of select important *E. coli* sero-groups

Among the sero-groups evaluated (026, 045, 0103, 0126, and 0146), only three sero-groups were detected in this study and included 026, 0103 and 0146 (Table 2). In total 24 isolates belonged to these three sero-groups with the most common being 026 [18] while [4] belonged to 0103 and the other [2] belonged to the 0146 sero-group. Interestingly, all the isolates were detected in young goat kids less than six months and especially around the weaning period. Of the 24, twenty-two [21] were detected around the weaning period (0-7DPW). Two [2] of the 026 isolates were detected in the diarrhea group while the rest were detected in apparently healthy goat kids. All except one of the isolates harbored at least one virulence gene and the virulence gene profiles and the phylogenetic groups for the other isolates are shown in Additional file 2. Most of the isolates were *stx1/stx2/hly/eaeA* positive (23%) while 12% had only the *hly* gene detected. The other combination of genes was detected in less than 10% of the isolates. Of these isolates, 75% belonged to the D phylogroup, 21% to B2 and 4% to B1.

### Prevalence of virulence genes and phylogenetic grouping of *E. coli* isolates from diarrheic samples

**Fig. 5:**
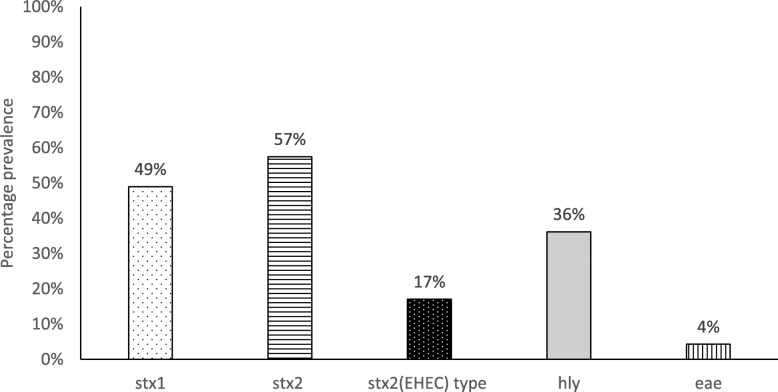
Prevalence of virulence genes in *E. coli* isolates from diarrheic animals

A total of 47 samples were isolated from diarrheic stools from young goats during the study. The stools were either collected from kids before weaning (17 isolates) or at day of weaning 0DPW (4 isolates), 1DPW [12] or 2DPW [14]. Most diarrheic stools were detected during the weaning period. The prevalence of the virulence genes in this cohort was *stx1* (49%), *stx2* variant (57%), *stx2* (17%), *hly* (36%) and *eaeA* (4%) (Fig. 5). The most significance finding in this group was the relatively higher prevalence of both the EHEC type *stx2* genes and the other *Stx2* genes which mirrored the prevalence detected in the healthy peri-weaning group (15 and 54% respectively). Nine different virulence profiles were detected in the positive isolates from this group. Similar to the peri-weaning group, most isolates were of the *stx1/stx2/hly* virulence profile (35%), *stx1/stx2* isolates were 16%, while *stx1* and *stx2* isolates were each 13% (Additional file 1). Isolates from diarrheic goats belonged to three phylogenetic groups; A, B1 and D (Table 2). Overall, group D was the most common (74%) in isolates that were positive for virulence genes irrespective of virulence profiles followed by group B1 23% (Additional file 1). We detected a significantly (*P* = 0.05) higher percentage of D phylogenetic group (74%) *E. coli* isolates in the diarrhea group compared to the other age matched healthy littermates six months and below (59%).

## Discussion

*Escherichia coli* is a normal inhabitant of the mammalian gut but some strains have the ability to cause enteric and extra-intestinal diseases in both animals and human. Few studies have evaluated or reported the diversity and virulence characteristics of *E. coli* isolates in goats. Moreover only one study that involved longitudinal characterization of *E.coli* isolates in goats was found in literature [25].

In this study, all the four virulence genes (*stx1*, *stx2* (EHEC), *stx2*, *eaeA* and *hly*) evaluated were detected in at least one isolate either singly or in combination. Overall, virulence genes and STEC were detected in isolates from goat kids in higher proportions than adult animals. Additionally, isolates with 2 or more virulence genes were significantly higher in pre-weaned and goat kids around weaning than in adult goats. Most isolates harbored either the *stx1/stx2/hly* or *stx1/hly* virulence profile. This is similar to findings reported in dairy goats in Spain [21]. Isolates with a single gene (*hly*) were more frequently detected in isolates from adult goats than young goat kids. We also found that isolates harboring *Stx1* and Hly were present in all age groups beginning as early as three weeks of age but the prevalence varied in different age groups. These results may imply that *E. coli* harboring these genes are adapted to colonizing the gastrointestinal gut of healthy meat goats and may persist in the animal gut once established. This is in agreement with previous studies, in dairy goats in Spain where they found STEC in goat kids as early as the first week of life [25] as well as other age groups including adult goats [25, 31]. Another study also found *E.coli* harboring virulence genes in goat milk and goat environment [35] and in Germany 75% of adult goats were found to harbor *Stx1* positive *E.coli* which was higher than the other farm species in the study. In Iran virulence genes were detected in slaughter age goats [26] and also in US slaughter age goats [27].

Our study found a significantly higher prevalence of the *stx1* and *eaeA* virulence genes in *E. coli* isolates from young nursing goat kids compared to their respective cohort of mothers grazing alongside them. Even for *hly* and *stx2 genes*, although not significantly, the pattern was similar with higher proportions of isolates harboring the virulence genes in goat kids than in adults. Results from the goat kid cohort that was followed from 3 weeks of age to one year indicate a clear pattern where the prevalence of the virulence genes *stx1*, *hly* and *stx2* peaked at weaning and then decreased over time reaching significantly lower levels in the goats at one year of age. Furthermore, we report novel findings that indicated a significant increase and the highest number of isolates with *stx1, hly* and *stx2* virulence genes around the weaning. Our findings are similar to those reported by other groups who found a higher prevalence of shiga toxin producing *E.coli* in calves than adult cattle [36–40] and in piglets compared to sows in Poland [41]. In contrast, our findings differ from previous studies in goats that reported a higher prevalence of virulence genes in samples from adult and replacement animals than in young animals [21, 25, 31] . These differences in the findings could be due to a combination of factors that include the method of sampling (one time vs longitudinal study) [21, 31], geographical location (Europe vs US) [21, 25, 31], age of animals included (4 weeks old goat kids [31] vs up to 13 weeks in in this study) and breed type (dairy goats vs meat goats in this study) [31] as well as management differences (artificial rearing vs nursing goat kids on pasture in this study) [25]. In one of these studies, differences in the age of first detection of Shiga toxin producing *E.coli* in young goat kids between the two farms that were followed over time was also reported [25] further indicating that several factors may affect the prevalence of virulence genes in goats. In cattle, a higher prevalence of shiga-toxin *E.coli* was reported in heifers than adult animals [42]. In the latter study, as animals increased in age after reaching maturity, the prevalence of virulence genes tended to decrease similar to findings in our study. The current study also found *Stx1/Hly* genes as the most common combination in all age groups which is similar to other studies in cattle that also found that *E. coli* isolates with *Stx1/Hly* virulence genes were common in all age groups [43]. While strains of *E. coli* having *Stx1* and *Hly* virulence genes alone are commonly found in healthy ruminants, the significance of these isolates in animals has not fully been explored. However, sero-groups O9, O22, O26, O103 and O105, that only possess these two virulence genes have been associated with human uraemic syndrome and have been isolated from healthy bovine species [44].

The enterocyte attaching and effacing (*eaeA)* gene protein (intimin) remains one of the important virulence factors that EPEC and STEC *E.coli* utilizes to cause intestinal pathology in both humans and animals [45, 46]. In humans, EPEC strains cause diarrhea in young children and susceptibility is known to decline with age [7]. In a rabbit experimental model, the *eaeA* gene was required for colonization and pathology in the intestines by O157: H7 *E.coli* [47]. Shiga toxin producing *E.coli* that produce intimin are associated with hemorrhagic diarrhea and hemolytic uremic syndrome in humans although non-O157 STEC *E.coli* without the *eaeA* gene have also caused hemorrhagic uremic syndrome in outbreaks in the US and Australia [48, 49]. In the current study, *E. coli* with the intimin protein coding gene (*eaeA)* were most prevalent in healthy pre-weaned meat goats with the highest prevalence (70%) being detected in healthy 3-week goat kids and declined as the kids grew older. Isolates with only eaeA gene (EPEC) were detected in 16% of pre-weaned goat kids. At one year of age, no *eaeA* gene was detected in any of the *E. coli* isolates. Similarly, other groups studying *E.coli* isolates from dairy cattle and dairy goats found that *E.coli* strains from calves [38] and goat kids [31] had a higher prevalence of *eaeA* gene than from adult dairy cattle and adult goats respectively. In a previous study, EPEC were suggested to play a role in diarrhea in goat kids and lambs [50]. Only a few isolates from diarrheic goats in this study possessed the *eaeA* gene. In agreement with our study, *eaeA* was detected in both healthy and diarrheic calves [51–53] with a higher prevalence in healthy calves compared to diarrheic calves [53]. Other previous studies in cattle, sheep and goats have reported similar prevalence of the *eaeA* gene in *E. coli* isolates from healthy and diarrheic animals. In a recent study characterizing virulence genes in *E. coli* isolates from diarrheic sheep, goats and calves, *eaeA* was not detected in isolates from sheep and goats [22] except from calves. Failure to detect high prevalence of the *eaeA* gene in isolates from diarrheic samples in this and previous studies may imply that *eaeA* gene protein is not required for *E. coli* colonization and disease pathology in the ruminant gut. Alternatively, it may be due to the fact that no direct *E. coli* diarrhea causal relationship was determined in these studies or age played a role in sheep and goats since EPEC are mostly associated in diarrhea in young animals. It is also possible that other microorganisms (viruses, parasites) may have been responsible for diarrhea in these animals as some studies also detected rotaviruses, coronaviruses, *enterococcus* and also *Salmonella spp* in the same animals [53]. In our present study, most of the diarrheic animals were detected in older kids (around 3 months) and were also parasitized by coccidia parasites and worms and detailed diagnostics to establish the exact cause of diarrhea was not pursued. Whether the *eaeA* positive *E. coli* isolates in healthy young goat kids detected in this study are potential pathogens in goats or humans remain to be evaluated. It may be that these isolates are potential pathogenic strains in very young animals that are kept in check by immune or bioactive molecules secreted in the gut by other beneficial gut microbes as has been reported for *Bifidobacteria* species in humans [54, 55] and/or produced from the mother’s milk. In this case any stress that cause gut dysbiosis may therefore result in these isolates inducing disease in these animals.

Another important *E. coli* virulence gene evaluated was the *Stx2* the product of which is the shiga toxin 2. This factor is known to be of higher virulence than the shiga toxin 1 and play a significant role in disease pathology causation in human. Seven variants (a-g) of the stx 2 factor are known to exist with some being more virulent in humans than others (reviewed in [7]). The presence of *Stx2* virulence genes in *E. coli* in ruminants has been reported in many other studies involving dairy goats, cattle and sheep [31] but the significance of the gene has not been fully described. Some studies in cattle found over 50 % of isolates from healthy animals carried the *Stx2* genes [44] while other studies in healthy goats also found over 70% of isolates harboring the *Stx2* gene [56]. Furthermore, none of the previous studies has evaluated the temporal pattern of the *stx2* gene in *E. coli* isolates from different age groups of ruminants. In this study we used two different *stx2* gene primers; one targeting the *stx2* (EHEC) gene found in *E.coli* isolates associated with the hemorrhagic uremic syndrome (HUS) [57] and another set used in a previous ruminant study [53]. The prevalence of the EHEC type *Stx2* virulence gene in the *E. coli* isolates from this cohort was relatively low compared to the other virulence genes evaluated. None of the *E. coli* isolates from goat kids prior to weaning, at 6 months, at one year or adult meat goats harbored this gene. Interestingly, the period around weaning was uniquely the only stage where this gene was detected in *E. coli* isolates from apparently healthy goat kids and isolates from diarrheic animals at weaning. No other study in the literature was found that had evaluated the temporal virulence gene detection in *E. coli* isolates from meat goats or other farm animals and focusing on weaning period specifically. On the other hand, using the primer set used in the ruminant study, this study detected *Stx2* gene in a large number of isolates from goat kids especially during weaning and also those from diarrheic animals compared to isolates from other age groups, a finding that has not been reported before. This finding calls for an understanding and further characterization of the *stx2* subtypes found in different farm animals, geographical regions and their clinical significance in these animals or in humans. Whether this pattern indicates a real significant role of the Stx2 in *E. coli* colonization of young diarrheic or stressed animals in meat goats remain to be further explored. In sheep, a higher number of shiga toxin *E.coli* (STEC) was reported in diarrheic lambs compared to healthy age matched lambs in the same study [6]. The exact reason for the pattern of higher detection of *stx2* genes around weaning period is not clear. Whether the *Stx2* gene plays a role in virulence of *E. coli* isolates in ruminants has also not been fully understood. However, further characterization of the stx2 subtypes in goats need to be addressed to have clear understanding of the diversity and significance of these isolates. We postulate that the stress of weaning may have contributed to a reduced host immunity or altered gut homeostasis in these young goat kids. This may have made the gut environment favorable for proliferation of specific minor low copy numbers of *E. coli* strains that otherwise are unable to compete in normal healthy animals when stress is not a factor.

A number of *E.coli* sero-groups including the O157 and non O157 strains with and without the shiga toxin genes are of significance in both animal and public health (reviewed in [14]. Many of these serogroups have been found in the gut of healthy cattle and goats [27, 31, 44] underscoring the importance of determining serogroups of *E. coli* isolated from food animals including goats. Three sero-groups (O26, O103 and O146) of *E. coli* were detected in this study. Of note is that all the sero-groups strains detected in this study have been implicated either in diarrhea in ruminants (026) [58] or human infections [14, 17, 18]. These sero-groups were all interestingly detected around the weaning period in young goat kids. The finding again points to a possibility of stress playing a role in proliferation of important pathogenic *E. coli* strains. This is similar to findings reported in cattle [29, 30] for *E.coli* O157 strain. Furthermore in beef cattle, transitioning of beef calves from weaning to feedlot was also associated with an increased prevalence of important serogroups including 026, 0103, 045, 0121 and 0157 [59] again indicating a possible role of stress.

The phylogenetic diversity of *E. coli* isolates from goats is currently not fully understood. Furthermore, it is not known if any factors including age or stress play a role in diversity of the *E. coli* phylogenetic groups colonizing the gut of ruminants in general as this has not been reported anywhere in the literature. A few studies have reported the phylogenetic groups of *E.coli* from goats [26, 34, 60–62] and other animals [63–66] in which either fewer isolates were evaluated and/or the isolates were all from adult animals or the age was not reported. This study characterized the phylogenetic distribution of *E. coli* isolates in different age groups using a cohort study that was evaluated over a one-year period. In this study, we detected the four main phylogenetic groups in goats with significant differences in the predominant phylogenetic groups based on the age and also weaning stress. Young meat goats less than six months were predominantly hosts to group D *E. coli* but these were mostly isolated during the weaning period. Goats six months and older were predominantly hosts to group B1. Our study findings are similar to [26, 34, 60] who found a high prevalence of the B1 group in adult goats and a few isolates of A phylogenetic group. Although the prevalence in individual species was not mentioned, Derakhshandeh et al [64] also found a predominance of group D *E.coli* in ruminants that included goats. In adult lactating dairy cattle, group B1 was the predominant phylogenetic group detected followed by group A [67, 68]. Age also influenced the phylogenetic diversity of commensal *E.coli* in pigs where young piglets were mostly found to host phylogenetic group B1 and the sows group A [41]. This differs from our findings of group D being the predominant phylogenetic group in young meat goats and B1 in adults goats but may point out to the many factors including species, geographical location, and diet that are reported to influence phylogenetic diversity of *E. coli* isolates in the gut [63, 65, 66, 69]. Our study also detected group B2 *E. coli* isolates mostly in kids less than six months with the highest number of isolates being detected around the weaning period. In the previous studies evaluating *E.coli* isolates from goats [26, 34, 60, 61] only one reported a few isolates belonging to phylogenetic B2 [61]. *E. coli* isolates belonging to group A were very few in all age groups in this study. *E. coli* strains belonging to groups B2 and D are known to contain more virulence factors than groups A and B1 and also more commonly involved in extra-intestinal infections especially group B2 [32]. The intestinal pathogenic strains are mainly found in groups A, B1 and D (reviewed in [34]. Thus in this study we detected *E. coli* strains belonging to phylogenetic groups that would be considered mostly normal commensals (A and B1) and also those that are potential pathogens although the prevalence depended on the age of the animals. To our knowledge this is the first study to evaluate and report on phylogenetic diversity of commensal *E. coli* in different age groups in small ruminants.

## Conclusion

In this first of its kind study, we carried out a one-year longitudinal study using a cohort evaluating both the phylogenetic diversity of *E. coli* isolates from different age groups of pastured meat goats and also prevalence of virulence genes. We also evaluated the prevalence of select important sero-groups among the isolates detected from the different age groups. This study unraveled hitherto unreported unique patterns on phylogenetic diversity of *E. coli*, virulence gene and important serogroup prevalence patterns based on the age and growth phases in pastured meat goats. Additionally, we hereby report findings that some animal growth stages (weaning)/health status (diarrhea) probably due to their inherent stress inducing characteristics were associated with a higher prevalence of *E. coli* harboring important virulence genes and also isolates that belong to sero-groups and phylogenetic groups that are important to both animal and human health. Thus more attention in reducing prevalence of these strains needs to focus on reducing stress in these animals and paying close attention to pre-weaning and weaning period. The significance of the *E. coli* virulence genes and phylogenetic groups found to differ based on age or during weaning in the ruminant gut or in public health may need further studies.

## Methods

### Study animals and husbandry

The study used adult and goat kids of Spanish and Myotonic breeds belonging to Virginia State University (VSU), USA, research flock. These animals are predominantly maintained on pasture except for the dry season when animals are supplemented with hay as needed. The goat kids used in the study were born on the farm. The original Myotonic and Spanish does and bucks were brought to the farm over ten years ago and are bred yearly, selected and replaced to maintain the best genetics. For the current study, the nursing does and any other adult goats were all between one and half to six years old.

### Animal sampling

During 2017 kidding period, a group of twenty-five [25] newly born goat kids (pre-weaning kids) and nursing does (pre-weaning does) were randomly selected from the research flock and sampled to evaluate differences in diversity and virulence gene characteristics of *E. coli* isolates from growing goat kids and the respective nursing adult goats. Sampling started at the age of three weeks for the goat kids with subsequent samplings done monthly until the kids were three months old and ready for weaning. Around weaning, the kids were sampled at day of weaning (0DPW), one day after (1DPW), two days after (2DPW) and one week after weaning (7 DPW) (peri-weaning kids). Subsequently, the goat kids were sampled at six months (6 months) and at one year of age (yearling). The six months and one-year sampling group included the initial cohort of randomly selected goat kids (pre-weaning kids) and other littermates born at the same time, on the same research farm and maintained under the same management throughout the study. Inclusion of all littermates was done for increased statistical power. The adult goats were sampled during the pre-weaning period (pre-weaning does) and also when the goat kids were sampled at six months of age (adults). For phylogenetic characterization, additional 22 isolates from adults in the same farm from a previous study were also included. Additionally, fecal samples were collected from any goat kid that developed diarrhea during the study period. None of the animals were sacrificed during the study and at the end of the study they remained part of the research herd at Virginia State University. The study was approved by the Virginia State University Institutional Animal Care and Use committee under the Protocol (AACUC protocol 2017–01).

### Fecal sample processing

Fecal samples were collected from individual goats per rectum using well lubricated gloves and immediately transported to the laboratory in ice for *E. coli* isolation. At each sampling points, kids with diarrhea were sampled separately and the fecal samples stored separately. The samples were processed the same day. Laboratory processing of the samples for *E. coli* isolation, confirmation and storage followed a previously described protocol [62]. *E. coli* ATCC 25922 was used as a positive control. Confirmed *E. coli* isolates were preserved in 20% glycerol at − 20 °C or below pending processing for virulence genes, phylogenetic group determination and sero-groups.

### DNA extraction protocol

DNA from each *E. coli* isolate was extracted from overnight culture in Luria broth grown at 37C. The DNA extraction protocol followed a simple boiling method [70] with a few modifications as described previously [62]. The concentration and purity of the extracted DNA was determined using a Nanodrop 2000c and samples stored at -20C or below until further processing for determination of virulence genes, phylogenetic grouping and sero-groups.

### Detection of virulence genes and sero-groups

Detection of virulence genes *stx1*, *stx2*, *eaeA* and *hly* in the *E.coli* isolates was done using PCR and primers previously described in two studies [57, 71] using conventional PCR (*stx1, stx2, eaeA* and *hly*) and also SYBR green real time detection protocol (*Stx1*,*stx2* and *eaeA* (Table 3). One Stx2 primer targeted the *E.coli* 0157: H7 *stx2* gene (enterohemorrhagic *Escherichia coli (*EHEC) [57] while the other primer targeted a *stx2* gene previously detected in ruminants (*stx2*) [53, 71]. *E. coli* ATCC 35150 was used as a positive control strains for the virulence genes while water was used as negative control. The amplified products were visualized under UV light in a gel imager after separation in 2% ethidium bromide containing agarose gel. For SYBR green real time detection a cycle threshold of 30 and melting curve analysis was used to confirm the presence of the genes.

**Table 3:** Primers used in the study

Fragment size (bp)	Target gene	Sequence (5′-3′)	Primer	Ref
338	*Stx1*	TCTCAGTGGGCGTTCTTATG	Stx1-a	[57]
		TACCCCCTCAACTGCTAATA	Stx1-b	
115	*Stx2*(EHEC)	GCGGTTTTATTTGCATTAGC	Stx2-a	[57]
		TCCCGTCAACCTTCACTGTA	Stx2-b	
248	*eaeA*	ATGCTTAGTGCTGGTTTAGG	EAEA-a	[57]
		GCCTTCATCATTTCGCTTTC	EAEA-b	
569	*hlyA*	AGCTGCAAGTGCGGGTCTG	HlyA-a	[57]
		TACGGGTTATGCCTGCAAGTTCAC	HlyA-b	
152	*O26wzx*	GCGCTGCAATTGCTTATGTA	Wzx-F	[72]
		TTTCCCCGCAATTTATTCAG	Wzx-R	
527	*O45wzx*	CCGGGTTTCGATTTGTGAAGGTTG	Wzx-F	[72]
		CACAACAGCCACTACTAGGCAGAA	Wzx-R	
321	*O103wzx*	TTGGAGCGTTAACTGGACCT	Wzx-F	[72]
		GCTCCCGAGCACGTATAAG	Wzx-R	
925	*O126wzx*	TTAGCTCTCGTAGAGGCTGGTGTT	Wzx-F	[72]
		ATGTCATTCCTGGGACGCGAATGT	Wzx-R	
640	*O146wzx*	AGGGTGACCATCAACACACTTGGA	wzx-F	[72]
		AGTTCAATACTGTCGCAGCTCCTC	wzx-R	
118	*Stx2*	GTGCCTGTTACTGGGTTTTTCTTC	Stx2F	[53]
		AGGGGTCGATATCTCTGTCC	Stx2R	
211	*yjaA*	TGAAGTGTCAGGAGACGCTG	YjaA.1	[32]
		ATGGAGAATGCGTTCCTCAAC	YjaA.2	
152	*tspE4.C2*	GAGTAATGTCGGGGCATTCA	TspE4C2.1	[32]
		CGCGCCAACAAAGTATTACG	TspE4C2.2	
279	*chuA*	GACGAACCA ACGGTCAGGAT	ChuA.1	[32]
		TGCCGCCAGTACC AAAGACA	ChuA.2	
401	*E. coli* 16 s	CCCCCTGGACGAAGACTGAC	EC16S-a	[57]
		ACCGCTGGCAACAAAGGATA	EC16S-b	

Similarly, O sero-group primers specific for the conserved part of the O antigen gene (*wzx* gene) [72] (Table 3) were used for screening for the presence of select important non-O157 sero-groups (O146, O126, O45, O103, and O26) using conventional PCR followed by visualization under UV light in 2% gel. These sero-groups were chosen based on their reported association with either diarrhea in food animals and/or food safety in human and also recent detection in goats at slaughter.

### Characterization of phylogenetic groups

Each confirmed *E.coli* isolate was subjected to a multiplex PCR of the genes *chuA*, *yjaA* and DNA fragment *TspE4.C2* following the previously described protocol [32] and the primers (Table 3) to determine the phylogenetic grouping. The amplified products were electrophoresed in a 2% ethidium bromide agarose gel and visualized under UV light in a gel imager. The isolates were grouped in to either A, B1, B2 or D groups based on the presence or absence of the three genes as previously described [32].

### Statistical analysis

The proportions of isolates with evaluated traits (virulence genes, phylogenetic groups) were determined for all age groups. Each individual isolate was considered the unit of analysis. The differences in proportion of isolates with each trait among age groups were tested using the MedCalc N-1 Chi-squared Comparison of proportions calculator 2020 [73]. Statistical comparisons were considered significant at *P* < 0.05 (Table 4).

**Table 4:** Statistical results of comparisons of proportions of virulence genes and phylogenetic groups of isolates analyzed using MedCalc Software

Comparison groups	Chi square value	***P*** value
Pre-weaned kids vs nursing does *stx1*	13.803	P = 0.0002
Pre-weaned kids vs nursing does *stx2*	3.160	*P* = 0.0755
Pre-weaned kids vs nursing does *eaeA*	18.394	P < 0.0001
Pre-weaned kids vs nursing does *hly*	2.813	*P* = 0.0935
Kids 0DPW vs kids 1DPW *Stx1*	14.5	P = 0.0001
Kids 0DPW vs kids 2DPW *Stx1*	11.5	P = 0.0007
Kids 0DPW vs kids 7DPW *Stx1*	6.8	P = 0.009
Kids 0DPW vs kids 1DPW *hly*	15.2	P = 0.0001
Kids 0DPW vs kids 2DPW *hly*	24.5	P < 0.0001
Kids 0DPW vs kids 7DPW *hly*	21.0	P < 0.0001
Preweaned and periweaning kids vs all adults *hly*	28.1	P < 0.0001
Preweaned and periweaning kids vs all adults STEC	41.4	P < 0.0001
Preweaned and periweaning kids vs all adults all virulence genes	25.4	P < 0.0001
Preweaned and periweaning kids vs all adults 2 or more virulence genes	26.48	P < 0.0001
Group D phylogenetic group (diarrhea (kids) vs healthy littermates)	3.709	*P* = 0.0541
Group D phylogenetic group (goat kids vs adults)	27.480	P < 0.0001
Group D phylogenetic group (pre-weaning kid vs weaning period)	4.727	*P* = 0.0297
Group B phylogenetic group (adults vs goat kids)	20.860	P < 0.0001

## Supplementary information

**Additional file 1 **Table 1**.** Virulence gene profiles of isolates harboring virulence genes and their corresponding phylogenetic groups.

**Additional file 2 **Table 2**.** Virulence profiles and phylogenetic groups of sero-groups detected in the study.

## Data Availability

We have attempted to include all data generated and used to draw conclusions during the study in the manuscript but it is also available from the corresponding author upon request.

## Abbreviations

Stx: Shiga toxin
eaeA: Effacing and attaching
hly: Hemolysin
*E. coli*: *Escherichia coli*
EHEC: Enterohemorrhagic *Escherichia coli*
STEC: Shiga toxin producing *Escherichia coli*
DPW: Days post weaning
DNA: Deoxyribonucleic acid
PCR: Polymerase chain reaction
US: United States
*wzx*: The O antigen processing proteins, the Wzx (O antigen flippase)
